# QT Prolongation in Cancer Patients

**DOI:** 10.3389/fcvm.2021.613625

**Published:** 2021-02-25

**Authors:** Peter Kim, Luke Masha, Amanda Olson, Cezar Iliescu, Kaveh Karimzad, Saamir Hassan, Nicolas Palaskas, Jean-Bernard Durand, Cheuk Hong Leung, Juan Lopez-Mattei

**Affiliations:** ^1^Division of Internal Medicine, Department of Cardiology, The University of Texas MD Anderson Cancer Center, Houston, TX, United States; ^2^Department of Cardiology, Oregon Health and Science University, Portland, OR, United States; ^3^Department of Biostatistics, The University of Texas MD Anderson Cancer Center, Houston, TX, United States

**Keywords:** QT prolongation, cardiooncology, ECG, torsades de pointes, cardiac monitoring in clinical trials

## Abstract

**Background:** QT prolongation and torsades de pointes pose a major concern for cardiologists and oncologists. Although cancer patients are suspected to have prolonged QT intervals, this has not been investigated in a large population. The purpose of this study was to analyze the QT interval distribution in a cancer population and compare it to a non-cancer population in the same institution.

**Methods:** The study was a retrospective review of 82,410 ECGs performed in cancer patients (51.8% women and 48.2% men) and 775 ECGs performed in normal stem cell donors (47.9% women and 52.1% men) from January 2009 to December 2013 at the University of Texas MD Anderson Cancer Center. Pharmacy prescription data was also collected and analyzed during the same time period. Correction of the QT interval for the heart rate was performed using the Bazett and Fridericia formulas.

**Results:** After QT correction for heart rate by the Fridericia formula (QTcF), the mean and 99% percentile QTc for cancer patients were 414 and 473 ms, respectively. These were significantly longer than the normal stem cell donors, 407 and 458 ms, *p* < 0.001, respectively. Among the cancer patients, the QTc was longer in the inpatient setting when compared to both outpatient and emergency center areas. The most commonly prescribed QT prolonging medications identified were ondansetron and methadone.

**Conclusion:** Our study demonstrates significantly longer QTc intervals in cancer patients, especially in the inpatient setting. Frequently prescribed QT prolonging medications such as antiemetics and analgesics may have a causative role in QT prolongation seen in our cancer hospital.

## Introduction

Prolongation of the QT interval is a well-recognized risk factor for potentially life-threatening ventricular arrhythmias and sudden cardiac death (1). With the development of novel anticancer therapies, many new biologic, immunologic, and targeted agents have been shown to alter cardiac repolarization and prolong the QT interval. A classic example is arsenic trioxide, which is an effective agent used to treat acute promyelocytic leukemia—an otherwise fatal disease. In one study of such treatment, severe QT prolongation (greater than 500 ms) was noted in 40% of patients receiving arsenic (2). Commonly used tyrosine kinase inhibitors such as vorinostat, dasatinib, lapatinib, and nilotinib have also been associated with QT prolongation (3–6).

QT prolongation is an important consideration in drug development and regulation. In light of the potentially fatal outcome of severe QT prolongation in investigational agents, the US Food and Drug Administration recommends periodic QT monitoring (7). Postmarketing data have identified drug-induced QT prolongation as the most common indication for withdrawal of medications from the market (8). In addition to safety regulation, the increasing costs of preclinical drug development (9) has limited the viability of otherwise promising investigational agents. Some pharmaceutical companies allocate 22% of total initial phase 1 clinical costs to QT monitoring and with advancement to phase 2, those costs may increase 6-fold (9).

Population studies have been used to identify the normal ranges of QT intervals. Unfortunately, there has been a paucity of data in oncologic patients, and only a few studies have investigated the QT intervals and cardiac event distributions in these populations (10, 11). These studies are limited by small patient populations, but seem to suggest a different range of QTc in cancer patients. In one such study (11), 15% of cancer patients required premature discontinuation or exclusion from potential curative cancer therapy when QTc exclusion guidelines were applied because the QTc cutoffs were derived from healthy populations. Often, cancer patients who enroll in investigational drug studies have previously been treated with multiple cancer therapeutics and are receiving several concurrent medications including anti-emetics, which are known to prolong the QTc interval. This further limits the determination of an investigational drug's effect on cardiac repolarization. Additional confusion arises regarding the clinical significance of QTc prolongation in the cancer patient population. Currently, there is limited data on the incidence of QTc-associated serious cardiac events in cancer patients. Available data in the non-cancer population has yielded a wide range of incidence rates from as low as 2.5 serious events per million years (12) in some large observational studies to as high as 12.5% incidence with the initiation of certain anti-arrhythmic agents (13, 14).

The primary objective of our study was to describe the QT intervals in cancer patients and compare them with those of healthy stem cell donors.

## Methods

The study and methodology were reviewed and approved by the Institutional Review Board of The University of Texas MD Anderson Cancer Center.

### Study Population

For our primary objective, we collected the first performed electrocardiogram (ECG) for oncologic patients older than 18 years who were treated at The University of Texas MD Anderson Cancer Center from January 2009 to December 2013 in the emergency department, outpatient, and inpatient settings. By protocol, we excluded pediatric patients to limit the exposure of age-related QT interval differences. Additional exclusion criteria were any ECG findings that would limit accurate measurement of the QT interval, including the presence of a significant intraventricular conduction delay or a paced rhythm. We also collected the first available ECGs from healthy stem cell donors in the same time interval to serve as a comparison control. The QT intervals were measured using an automated computerized ECG analysis algorithm and then confirmed manually by an interpreting cardiologist.

Standard 12-lead ECGs were obtained at 25 mm/s and 0.1 mV/mm on strips of lined paper. Digital ECG measurements and calculations were made using the hospital Cardiac Science ECG system. The QT interval was defined as the first reflection of the QRS complex to the return of the T wave to the isoelectric line, excluding the U wave. The computer analysis selected the longest QT interval from the lead that had a clear QRS complex and T wave. All ECG measurements were evaluated and manually confirmed by a cardiologist. The QT interval was corrected for heart rate variation using both the Bazett (QTcB = QT/√RR) and Fridericia (QTcF = QT/^3^√RR) formulas.

Medication prescription data was also collected during the same time interval. Both inpatient and outpatient pharmacy queries were performed and the most frequently prescribed medications were obtained for review.

### Statistical Analysis

Mean values with standard deviations (SDs) were given for continuous data. Frequency statistics were provided for categorical data. Differences in continuous variables and categorical variables between two groups were assessed by two-sample *T*-tests and Chi-Squared tests, respectively. ANOVA tests with Bonferroni correction were used to compare continuous variables between multiple groups. Statistical significance was set at a two-tailed probability level <0.05 for all analyses. A Bland and Altman plot was performed to compare differences in QTcB and QTcF against the mean of QTcB and QTcF among cancer patients at different heart rate ranges. Statistical analyses were performed using STATA 14.2 software (StataCorp, College Station, TX) and R 3.3.1.

The authors had full access to the data and take responsibility for its integrity. All authors have read and agree to the manuscript as written.

## Results

We identified 221,332 ECGs performed in cancer patients and stem cell donors from January 2009 to December 2013. After exclusion criteria were applied, 82,410 first reported ECGs performed in cancer patients were selected and 775 ECGs performed in healthy stem cell donors remained for analysis. The baseline demographics of these two groups are shown in Table 1.

**Table 1:** Characteristics of cancer patients and healthy stem cell donors.

**Characteristic**	**Cancer patients (*n* = 82,410)**	**Healthy stem cell donors (*n* = 775)**	***P*-value**
Age, mean (SD), y	59.1 (13.5)	47.1 (14.3)	<0.001
Men, %	48.2	52.1	0.03
Number of ECGs performed	82,410	775	
HR, mean (SD), bpm	74.1 (17.0)	66.1 (11.7)	<0.001
QRS duration, mean (SD), ms	89.2 (10.3)	90.7 (9.58)	<0.001
Bazett QTc, ms			
Mean (SD)	427 (23.9)	413 (23.4)	<0.001
99th percentile	491	468	<0.001
QTc ≥ 450	12,933 (15.7%)	47 (6.1%)	<0.001
QTc > 500	163 (0.2%)	0 (0%)	0.412
Fridericia QTc, ms			
Mean (SD)	414 (22.1)	407 (19.9)	<0.001
99th percentile	473	458	<0.001
QTc ≥ 450	4,513 (5.5%)	22 (2.8%)	0.001
QTc > 500	53 (0.06%)	0 (0%)	1.00

*HR, heart rate*.

The mean QTcB and QTcF values were significantly higher for the cancer patient population than for the donor control population (427 vs. 413 ms, *p* < 0.001; 414 vs. 407 ms, *p* < 0.001, Table 1), respectively. The 99th percentile for QTcB and QTcF for the cancer patients were also significantly higher than the donor control population (491 vs. 468 ms, *p* < 0.001; 473 vs. 458 ms, *p* < 0.001). In addition, there were a greater number of patients with QTcB and QTcF values greater than or equal to 450 ms in the cancer patients. The percentage of cancer patients with QTcB ≥ 450 ms was 15.7 vs. 6.1% in the control patients. This was also reflected by QTcF although to a lesser degree, 5.5 vs. 2.8%, respectively.

The mean QTcB from ECGs performed in the inpatient setting were higher than those from ECGs obtained from outpatient clinics and the emergency department (430 vs. 426 ms, *p* < 0.001; 430 vs. 423 ms, *p* < 0.001, Table 2). The same analyses were performed on QTcF among the cancer patient population showing similar results.

**Table 2:** ECG characteristics of cancer patients.

**Group**	**Bazett QTc, ms**				**Fridericia QTc, ms**			
	**99th percentile**	***p***-value^*^	**Mean (SD)**	***p***-value^*^	**99th percentile**	***p***-value^*^	**Mean (SD)**	***p***-value^*^
All patients		0.608		<0.001		0.548		<0.001
Women	491		430 (22.7)		472		416 (21.8)	
Men	491		423 (24.6)		473		411 (22.2)	
Heart rate		<0.001		<0.001		<0.001		<0.001
60-80 bpm	487		426 (21.8)		475		416 (20.9)	
81-100 bpm	495		437 (21.3)		462		410 (20.1)	
Age								
(1) ≤ 30 y	488	(1)–(2) (*p* = 1.00)	422 (25.7)	(1)–(2) (*p* < 0.001)	459	(1)–(2) (*p* = 0.003)	404 (22.6)	(1)–(2) (*p* < 0.001)
(2) 31–60 y	489	(1)–(3) (*p* = 0.147)	426 (23.5)	(1)–(3) (*p* < 0.001)	468	(1)–(3) (*p* < 0.001)	412 (21.2)	(1)–(3) (*p* < 0.001)
(3) >60 y	493	(2)–(3) (*p* < 0.001)	428 (24.0)	(2)–(3) (*p* < 0.001)	476	(2)–(3) (*p* < 0.001)	416 (22.5)	(2)–(3) (*p* < 0.001)
Clinic setting								
(1) Outpatient	488	(1)–(2) (*p* < 0.001)	426 (23.5)	(1)–(2) (*p* < 0.001)	472	(1)–(2) (*p* = 0.004)	414 (21.6)	(1)–(2) (*p* < 0.001)
(2) Inpatient	497	(1)–(3) (*p* = 0.017)	430 (25.7)	(1)–(3) (*p* < 0.001)	476	(1)–(3) (*p* = 0.009)	412 (24.5)	(1)–(3) (*p* = 0.230)
(3) Emergency department	484	(2)–(3) (*p* < 0.001)	423 (22.6)	(2)–(3) (*p* < 0.001)	467	(2)–(3) (*p* < 0.001)	413 (20.6)	(2)–(3) (*p* < 0.001)

* *Permutation test comparing 99th percentiles; pairwise permutation test with Bonferroni adjustment*.

** *T-test comparing means; pairwise t-test with Bonferroni adjustment*.

**Figure 1:**
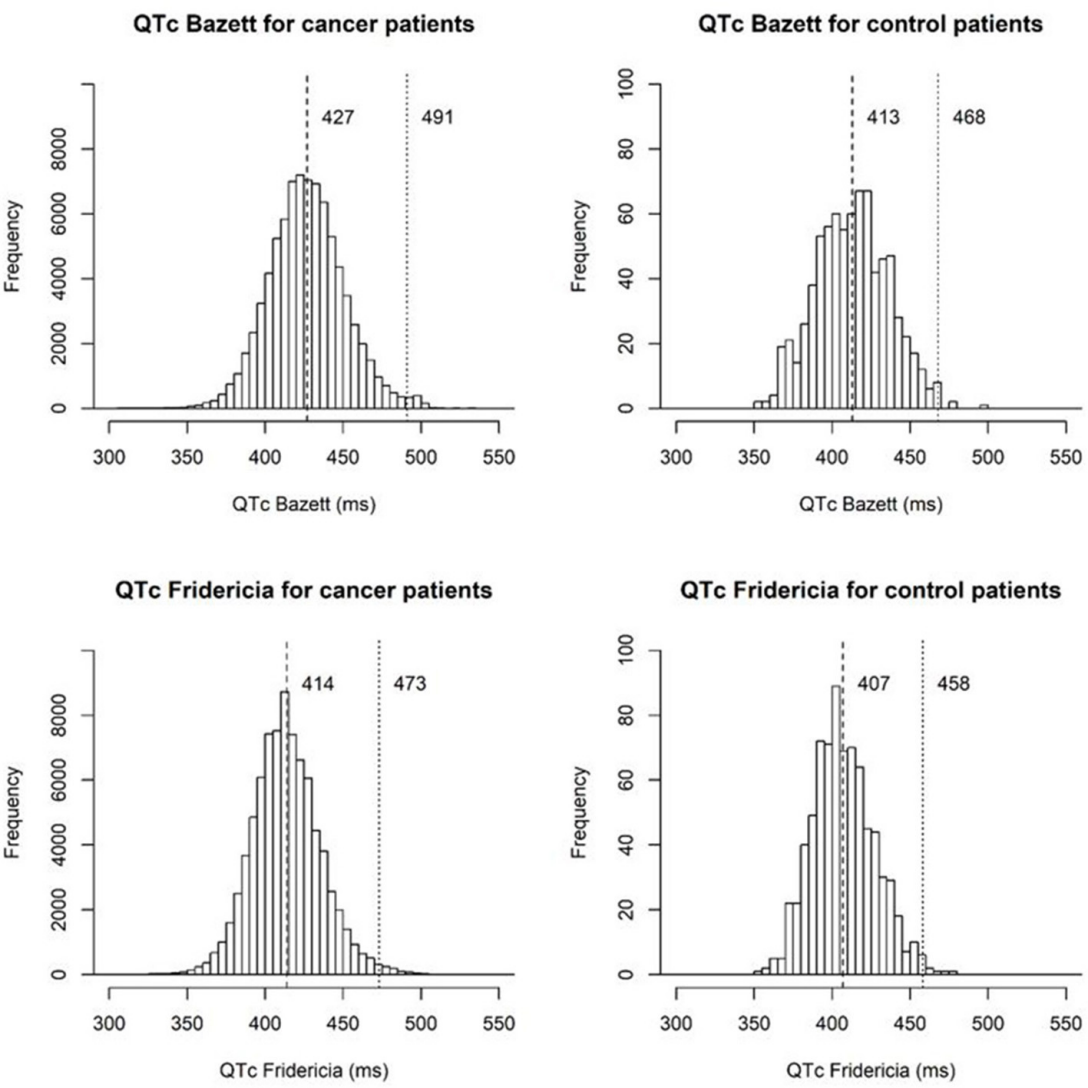
Histograms of QTc intervals by the Bazett and Fridericia formulas for cancer patients and stem cell donor controls marking the mean and 99th percentile QT values.

**Figure 2:**
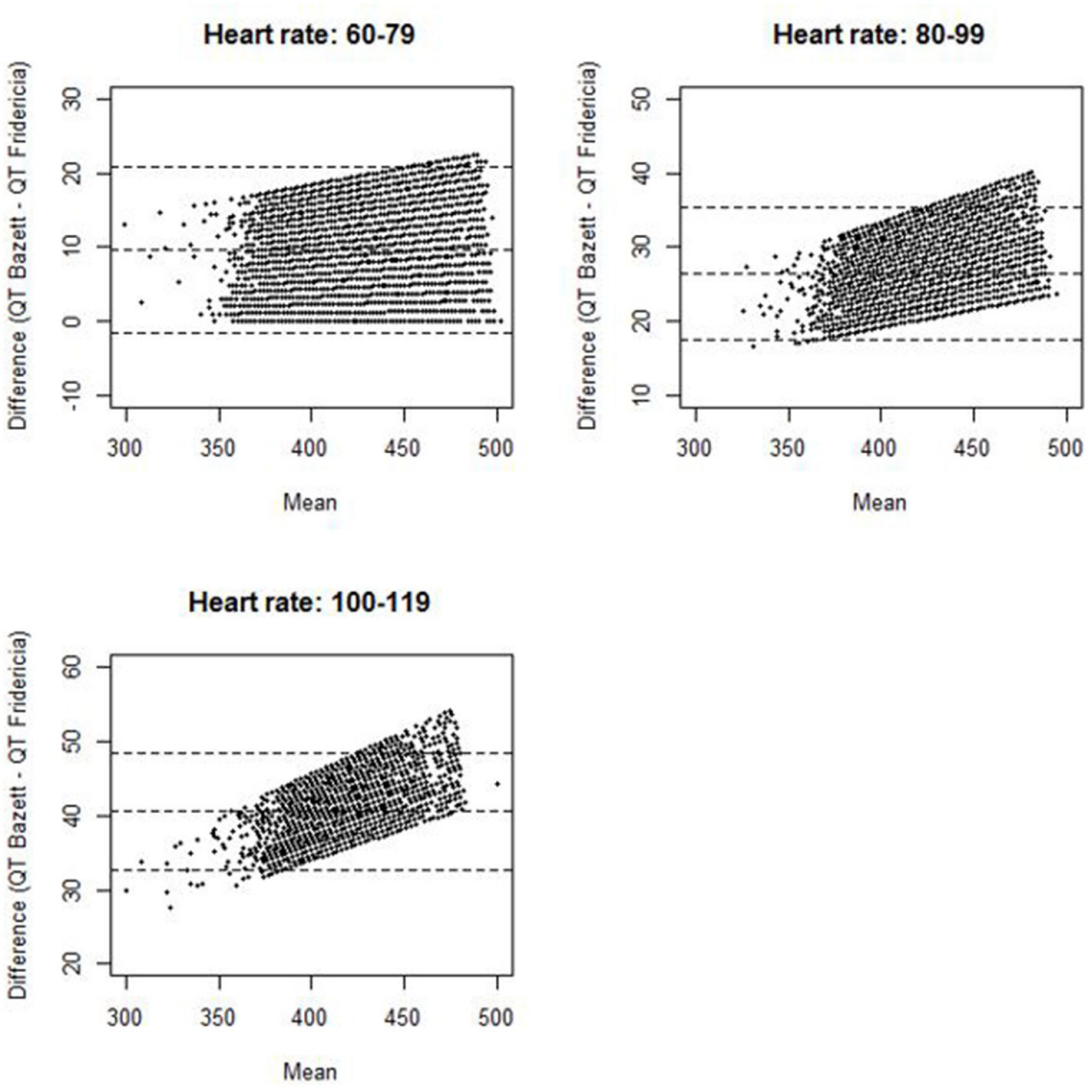
Bland and Altman plot for difference of QT Bazett and QT Fridericia against mean of QT Bazett and QT Fridericia among cancer patients stratified by heart rate (the dotted lines represent the upper limit of agreement, mean difference, and lower limit of agreement).

The distribution of QTc values by the Bazett and Fridericia formulas are shown in Figure 1, and comparisons between these formulas at different heart rates are shown in Table 2. The distribution of QTc intervals was a typical bell-shaped curve distribution. The difference of the QTcB and QTcF was compared against different heart rate ranges in a Bland and Altman plot which demonstrated higher values of QTcB compared to QTcF at heart rates > 100 bpm (Figure 2).

The pharmacy prescription data was collected and segregated between inpatient and outpatient pharmacies as shown in Table 3.

**Table 3:** Medications prescribed from 1/1/2009 to 12/31/2013.

**Inpatient Medications**		**Outpatient Pharmacy Prescriptions**	
**Medication**	**Doses**	**Medication**	**Doses**
Magnesium Sulfate	278,544,448	Acetaminophen/Hydrocodone	10,443,662
Ondansetron HCl	245,888,164	Xyloxylin	6,662,270
Dextrose	208,604,526	Sucralfate	3,979,366
Diphenhydramine	155,038,997	Hydromorphone	3,491,924
Acetaminophen	107,456,448	Oxycodone	3,445,530
Hydromorphone	94,697,820	Morphine	2,625,967
Dexamethasone Sodium Phosphate	84,320,780	Gabapentin	2,178,045
Heparin Sodium (Porcine)	81,200,126	Lactulose	1,958,813
Morphine	63,157,890	Methadone	1,821,234
Enoxaparin	31,922,397	Heparin Sodium (Porcine)	1,802,220
Acetaminophen/Hydrocodone	20,463,002	Metoclopramide	1,786,597
Pantoprazole Sodium	16,501,050	Docusate Sodium	1,744,570
Metoprolol Tartrate	12,775,562	Nystatin	1,557,765
Vancomycin HCl	10,002,811	Sennosides-Docusate Sodium	1,522,647
Piperacillin/Tazobactam	8,204,540	Sennosides	1,452,763
Cefepime HCL	7,090,713	Ondansetron HCl	1,407,213
Sodium Bicarbonate-Sodium Chloride	2,512,896	Magnesium Oxide	1,370,704
Sennosides-Docusate Sodium	2,235,408	Dexamethasone	1,282,392
Valacyclovir HCL	1,847,692	Tramadol HCl	1,256,464

*Green, No known risk of QT prolongation*.

*Yellow, Conditional/Possible risk of QT prolongation*.

*Red, Known risk of QT prolongation*.

## Discussion

Our results demonstrate that cancer patients have significantly prolonged QTc intervals compared with individuals without cancer. The differences noted in historical healthy controls were consistent with our internal matched stem cell donor controls.

Epidemiologic surveys of healthy individuals (15–18) have established that QTcB is abnormally prolonged when it exceeds 450 ms in men and 460 ms in women. These same studies suggest a normal mean (SD) QTcB of 390 (20) ms. With these criteria, it is estimated that less than 1% of healthy individuals have abnormal QTc prolongation at baseline.

Similar epidemiologic data is scarce in the cancer patient population, but such data is of importance in guiding clinical management as well as the design and inclusion strategies for oncology clinical trials. Varterasian et al. described the QTc distribution in 128 patients with various malignancies being evaluated for inclusion in a clinical trial. The researchers found at baseline a mean (SD) QTc of 417 (27) ms, suggesting that ~15% of cancer patients would be excluded from clinical trials based on the presence of a borderline prolonged QTc (10). Sarapa et al. reported similar findings in a survey of 160 patients (11). The ICH E14 guidelines recommend excluding from early-phase clinical trials patients with a baseline QTc greater than 450 ms, especially those with concomitant risk factors for arrhythmias (7, 19). However, these QTc cutoffs have not been rigorously evaluated in the cancer patient population and have little clinical data to support their endorsement.

Our study is the largest epidemiologic study to date attempting to define the QTc spectrum in a cancer patient population. The mean (SD) QTcF was 414 (22.1) ms related to the largest peak of the Gaussian distribution curve. This finding suggests that the QTc distribution spectrum in the cancer patient population has a significant rightward shift compared with both historical non-cancer patient reports and our non-cancer stem cell donor control population. Approximately 5.5% of the cancer patients had a QTcF greater than 450 ms compared to only 2.8% in the stem cell donors. Although the 99th percentile for QTcB in published historical healthy controls of 450 ms was smaller than that of our stem cell donor control population (468 ms), there was a greater difference when compared with the 99th percentile of our cancer patient population (491 ms). This significant shift in the cancer patient population's baseline QTc can likely be explained by several contributing factors, including polypharmacy with concomitant QTc-prolonging medications, higher incidence of electrolyte abnormalities, advanced age, and associated cardiovascular disease. In addition, our analysis demonstrated that the Fridericia correction had less variability at higher heart rates, and confirmed the utility of QTcF correction over QTcB in our patient population.

Although we were unable to collect individual medication data on all of the included patients, we were able to collect pharmacy prescription throughout the institution in both inpatient and outpatient pharmacies as shown in Table 3. The second most commonly administered inpatient medication was ondansetron which is known to cause QT prolongation. Three other commonly prescribed inpatient medications (diphenhydramine, pantoprazole, and piperacillin/tazobactam) also had conditional or possible risk of QT prolongation. Among the commonly prescribed outpatient prescriptions, two medications had known QT prolongation (methadone and ondansetron) and two had conditional or possible QT prolongation risks (metoclopramide and tramadol). The common use of these medications may be related to the differences of QT prolongation seen in the inpatient and outpatient ECGs.

Our findings raise several important concerns and questions in the observation of cancer patients' risk of arrhythmic events. Compared with both historical controls and our own cancer-free stem cell donors, QT intervals in our cancer patients were significantly elevated with noticeably higher QTc in the inpatient setting. The translation of longer QTc in cancer patients into clinical events needs further investigation. Also, the exact mechanism of this high incidence of QT prolongation is probably multifactorial and not well-understood. Although our pharmacy prescription data suggests this could be partly related to several QT prolonging medications, additional analysis of risk factors, including electrolyte imbalance, structural heart disease, and pre-existing ischemic heart disease will be needed to elucidate risk factors. Alternatives strategies to pain management and emesis control should be considered to lower the risk of prolonged QTc, as the use of methadone and ondasetron was quite prevalent.

The limitations to our study include its retrospective data collection and possible referral bias. Although QT intervals can be influenced by age, gender, certain medications, electrolyte imbalances, and structural heart disease, the purpose of this study was not to account for all individual confounding variables, but to rather describe the QT interval distribution in a generalized cancer population. Also, the differences in QTc were not compared to clinical endpoints such as ventricular arrhythmias and sudden cardiac death which would be an area of future research.

## Conclusion

Our study shows that QTc prolongation is more common in the cancer patient population, particularly in the inpatient setting than in previously reported healthy historical models. Drug prescription patterns for pain and emesis control might be associated with these findings. The association of serious arrhythmic events related to QT prolongation needs to be investigated in cancer patients. Further study in the application of QT intervals and setting appropriate thresholds in the routine monitoring of cancer patients is needed to better risk stratify the potential harm of newer cancer therapies.

## Data Availability Statement

The raw data supporting the conclusions of this article will be made available by the authors, without undue reservation.

## Author Contributions

All authors listed have made a substantial, direct and intellectual contribution to the work, and approved it for publication.

## Conflict of Interest

The authors declare that the research was conducted in the absence of any commercial or financial relationships that could be construed as a potential conflict of interest.

## References

[1] Vandael E Vandenberk B Vandenberghe J Pincé H Willems R Foulon V. Incidence of Torsade de Pointes in a tertiary hospital population. Int J Cardiol. (2017) 243:511–5. 10.1016/j.ijcard.2017.05.072 28576628

[2] Soignet SL Frankel SR Douer D Tallman MS Kantarjian H Calleja E. United States multicenter study of arsenic trioxide in relapsed acute promyelocytic leukemia. J Clin Oncol. (2001) 19:3852–60. 10.1200/JCO.2001.19.18.3852 11559723

[3] Lynch DR Jr Washam JB Newby LK. QT interval prolongation and torsades de pointes in a patient undergoing treatment with vorinostat: a case report and review of the literature. Cardiol J. (2012) 19:434–8. 10.5603/CJ.2012.0078 22825908

[4] Johnson FM Agrawal S Burris H Rosen L Dhillon N Hong D. Phase 1 pharmacokinetic and drug-interaction study of dasatinib in patients with advanced solid tumors. Cancer. (2010) 116:1582–91. 10.1002/cncr.24927 20108303

[5] Burris HA III Taylor CW Jones SF Koch KM Versola MJ Arya N. A phase I and pharmacokinetic study of oral lapatinib administered once or twice daily in patients with solid malignancies. Clin Cancer Res. (2009) 15:6702–8. 10.1158/1078-0432.CCR-09-0369 19825948 PMC3232441

[6] Hazarika M Jiang X Liu Q Lee SL Ramchandani R Garnett C. Tasigna for chronic and accelerated phase Philadelphia chromosome–positive chronic myelogenous leukemia resistant to or intolerant of imatinib. Clin Cancer Res. (2008) 14:5325–31. 10.1158/1078-0432.CCR-08-0308 18765523

[7] Food and H.H.S. Drug Administration, International Conference on Harmonisation; guidance on E14 Clinical Evaluation of QT/QTc Interval Prolongation and Proarrhythmic Potential for Non-Antiarrhythmic Drugs; availability Notice. Fed Regist. (2005) 70:61134–5. 16237860

[8] Shah RR. Can pharmacogenetics help rescue drugs withdrawn from the market? Pharmacogenomics. (2006) 7:889–908. 10.2217/14622416.7.6.889 16981848

[9] Fermini B Fossa AA. The impact of drug-induced QT interval prolongation on drug discovery and development. Nat Rev Drug Discov. (2003) 2:439–47. 10.1038/nrd1108 12776219

[10] Varterasian M Meyer M Fingert H Radlowski D Asbury P Zhou X. Baseline heart rate-corrected QT and eligibility for clinical trials in oncology. J Clin Oncol. (2003) 21:3378–9. 10.1200/JCO.2003.99.104 12947082

[11] Sarapa N Huang X Varterasian M Fingert H. Risk management and eligibility criteria for QTc assessment in patients with advanced cancer. J Clin Oncol. (2005) 23:203s. 10.1200/jco.2005.23.16_suppl.3047

[12] Sarganas G Garbe E Klimpel A Hering RC Bronder E Haverkamp W. Epidemiology of symptomatic drug-induced long QT syndrome and Torsade de Pointes in Germany. Europace. (2014) 16:101–8. 10.1093/europace/eut214 23833046

[13] Darpo B. Spectrum of drugs prolonging QT interval and the incidence of torsades de pointes. Eur Heart J Suppl. (2001) 3:K70–80. 10.1016/S1520-765X(01)90009-4

[14] Murray KT. Ibutilide. Circulation. (1998) 97:493–7. 10.1161/01.CIR.97.5.493 9490245

[15] Kobza R Roos M Niggli B Abächerli R Lupi GA Frey F. Prevalence of long and short QT in a young population of 41,767 predominantly male Swiss conscripts. Heart Rhythm. (2009) 6:652–7. 10.1016/j.hrthm.2009.01.009 19303371

[16] Funada A Hayashi K Ino H Fujino N Uchiyama K Sakata K. Assessment of QT intervals and prevalence of short QT syndrome in Japan. Clin Cardiol. (2008) 31:270–4. 10.1002/clc.20208 18543308 PMC6653181

[17] Gallagher MM Magliano G Yap YG Padula M Morgia V Postorino C. Distribution and prognostic significance of QT intervals in the lowest half centile in 12,012 apparently healthy persons. Am J Cardiol. (2006) 98:933–5. 10.1016/j.amjcard.2006.04.035 16996877

[18] Anttonen O Junttila MJ Rissanen H Reunanen A Viitasalo M Huikuri HV. Prevalence and prognostic significance of short QT interval in a middle-aged Finnish population. Circulation. (2007) 116:714–20. 10.1161/CIRCULATIONAHA.106.676551 17679619

[19] Morganroth J Shah RR Scott JW. Evaluation and management of cardiac safety using the electrocardiogram in oncology clinical trials: focus on cardiac repolarization (QTc Interval). Clin Pharmacol Ther. (2010) 87:166–74. 10.1038/clpt.2009.214 20010556

